# Criticality in Pareto Optimal Grammars?

**DOI:** 10.3390/e22020165

**Published:** 2020-01-31

**Authors:** Luís F Seoane, Ricard Solé

**Affiliations:** 1 Instituto de Física Interdisciplinar y Sistemas Complejos IFISC (CSIC-UIB), Campus UIB, 07122 Palma de Mallorca, Spain; 2 ICREA-Complex Systems Lab, Universitat Pompeu Fabra (GRIB), Dr Aiguader 80, 08003 Barcelona, Spain; 3 Institut de Biologia Evolutiva, CSIC-UPF, Pg Maritim de la Barceloneta 37, 08003 Barcelona, Spain; 4 Santa Fe Institute, 1399 Hyde Park Road, Santa Fe, NM 87501, USA

**Keywords:** syntax, Pareto-optimality, bottleneck method, phase transitions, statistical mechanics

## Abstract

What are relevant levels of description when investigating human language? How are these levels connected to each other? Does one description yield smoothly into the next one such that different models lie naturally along a hierarchy containing each other? Or, instead, are there sharp transitions between one description and the next, such that to gain a little bit accuracy it is necessary to change our framework radically? Do different levels describe the same linguistic aspects with increasing (or decreasing) accuracy? Historically, answers to these questions were guided by intuition and resulted in subfields of study, from phonetics to syntax and semantics. Need for research at each level is acknowledged, but seldom are these different aspects brought together (with notable exceptions). Here, we propose a methodology to inspect empirical corpora systematically, and to extract from them, blindly, relevant phenomenological scales and interactions between them. Our methodology is rigorously grounded in information theory, multi-objective optimization, and statistical physics. Salient levels of linguistic description are readily interpretable in terms of energies, entropies, phase transitions, or criticality. Our results suggest a critical point in the description of human language, indicating that several complementary models are simultaneously necessary (and unavoidable) to describe it.

## 1. Introduction

**Figure 1:**
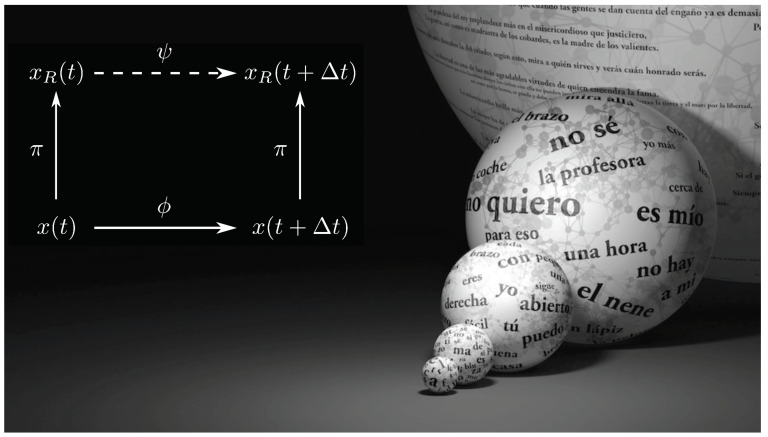
Different levels of grammar. Language contains several layers of complexity that can be gauged using different kinds of measures and are tied to different kinds of problems. The background picture summarizes the enormous combinatorial potential connecting different levels, from the alphabet (smaller sphere) to grammatically correct sentences (larger sphere). On top of this, it is possible to describe each layer by means of a coarse-grained symbolic dynamics approach. One particularly relevant level is the one associated to the way syntax allows generating grammatically correct strings x(t). As indicated in the left diagram, symbols succeed each other following some rules ϕ. A coarse-graining π groups up symbols in a series of classes such that the names of these classes; $x_{R}\left(t\right)$ also generate some symbolic dynamics whose rules are captured by ψ. How much information can the dynamics induced by ψ recover about the original dynamics induced by ϕ? Good choices of π and ψ will preserve as much information as possible despite being relatively simple.

What is the “right” level of description for the faculty of human language? What would allow us to properly describe how it operates given the multiple scales involved—from letters and words to whole sentences? This nested character of language organization (Figure 1) pervades the great challenge of understanding how it originated and how we could generate it artificially. The standard answer to these and similar questions is given by rules of thumb that have helped us, historically, to navigate the linguistic complexities. We have identified salient aspects (e.g., phonetics, formal grammars, etc.) to which whole fields are devoted. In adopting a level of description, we hope to encapsulate a helpful snippet of knowledge. To guide these choices we must broadly fulfill two goals: (i) the system under research (human language) must be somehow simplified and (ii) despite that simplification we must still capture as many relevant, predictive features about our system’s unfolding as possible. Some simplifications work better than others. In general, opting for a specific level does not mean that another one is not informative.

A successful approach to explore human language is through networks. Nodes of a language web can be letters, syllables, or words; links can represent co-occurrences, structural similarity, phonology, or syntactic or semantic relations [1,2,3,4,5,6,7]. Are these different levels of description nested parsimoniously into each other? Or do sharp transitions exist that establish clear phenomenological realms? Most of the network-level topological analyses suggest potential paths to understand linguistic processing and hint at deeper features of language organization. However, the connection between different levels are seldom explored, with few exceptions based on purely topological patterns [8]; or some ambitious attempts to integrate all linguistic scales from the evolutionary one to the production of phonemes [9,10].

In this paper, we present a methodology to tackle this problem in linguistics: When are different levels of description pertinent? When can we forgo some details and focus on others? For example, when do we need to attend to syntactic constraints, and when do we need to pay attention to phonology? How do the descriptions at different levels come together? This interplay can be far from trivial: note, e.g., how phonetics dictates the grammatical choice of the determiner form “a” or “an” in English. Similarly, phonetic choices with no grammatical consequence can evolve into rigid syntactic rules in the long term. Is the description at a higher level always grounded in all previous stages, or do descriptions exist that do not depend on details from other scales? Likely, these are not all or nothing question. Therefore, rather, how many details in a given description do we need to carry on to the next one?

To exemplify how these questions can be approached, we look at written corpora as symbolic series. There are many ways in which a written corpus can be considered a symbolic series. For example, we can study the succession of letters in a text. Then, the available vocabulary consists of all letters in the alphabet (often including punctuations marks):

(1) $$\begin{array}{ccc} \chi^{letters} & \equiv & \{a,b,\ldots ,z,!,?,\ldots \}. \end{array}$$

Alternatively, we can consider words as indivisible. In such cases, our vocabulary ($\chi^{words}$) would consist of all entries in a dictionary. We can study even simpler symbolic dynamics, e.g., if we group together all words of each given grammatical class and consider words within a class equal to each other. From this point of view, we do not gain much by keeping explicit words in our corpora. We can just substitute each one by its grammatical class, for example,

(2) $$\begin{array}{c} \mathrm{green}\;\;\mathrm{colorless}\;\;\mathrm{ideas}\;\;\mathrm{sleep}\;\;\mathrm{furiously}⟶adj\;\;adj\;\;noun\;\;verb\;\;adv. \end{array}$$

After this, we can study the resulting series that have as symbols elements of the coarse-grained vocabulary:

(3) $$\begin{array}{c} \chi^{grammar}\equiv \left\{noun,verb,adj,adv,prep,\ldots \right\}. \end{array}$$

Further abstractions are possible. For example, we can introduce a mapping that retains the difference between nouns and verbs, and groups all other words in an abstract third category:

(4) $$\begin{array}{c} \mathrm{adj}\;\;\mathrm{adj}\;\;\mathrm{noun}\;\;\mathrm{verb}\;\;\mathrm{adv}⟶cat_{3}\;\;cat_{3}\;\;noun\;\;verb\;\;cat_{3}. \end{array}$$

It is fair to ask which of these descriptions are more useful, when to stop our abstractions, whether different levels define complementary or redundant aspects of language, etc. Each of these descriptions introduces an operation that maps the most fine-grained vocabulary into less detailed ones, for example,

(5) $$\begin{array}{c} \pi :\chi^{words}\to \chi^{grammar}. \end{array}$$

To validate the accuracy of this mapping, we need a second element. At the most fundamental level, some unknown rules ϕ exist. They are the ones connecting words to each other in real language and correspond to the generative mechanisms that we would like to unravel. At the level coarse-grained by a mapping π, we can propose a description Ψ (Figure 1) that captures how the less-detailed dynamics advance. How well can we recover the original series depends on our choices of π and Ψ. Particularly good descriptions at different scales conform the answers to the questions raised above. The ϕ and Ψ mappings play roles similar to language grammar, i.e., sets of rules that tell us what words can follow each other. Some rules show up in actual corpora more often than others. Almost every sentence needs to deal with the Subject-Verb-Object (SVO) rule, but only seldom do we find all types of adjectives in a same phrase. If we would infer a grammar empirically by looking at English corpora, we could easily oversee that there is a rule for adjective order too. However, as it can be so easily missed, this might not be as important as SVO to understand how English works.

Here, we investigate grammars, or sets of rules, that are empirically derived from written corpora. We would like to study as many grammars as possible, and to evaluate numerically how well each of them works. In this approach, a wrong rule (e.g., one proposing that sentence order in English is VSO instead of SVO) would perform poorly and be readily discarded. It is more difficult to test descriptive grammars (e.g., a rule that dictates the adjective order), so instead we adopt abstract models that tell us the probability that classes of words follow each other. For example, in English, it is likely to find an adjective or a noun after a determiner, but it is unlikely to find a verb. Our approach is inspired by the information bottleneck method [11,12,13,14,15], rate distortion theory [16,17], and similar techniques [18,19,20,21,22]. In all these studies, arbitrary symbolic dynamics are divided into the observations up to a certain point, $\overset{\leftarrow }{x}$, the dynamics from that point onward, $\vec{x}$, and some coarse-grained model *R* (which plays the role of our π and Ψ combined) that attempts to conceptualize what has happened in $\overset{\leftarrow }{x}$ to predict what will happen in $\vec{x}$. This scheme allows us to quantify mathematically how good is a choice of R≡{π,Ψ}. For example, it is usual to search for models *R* that maximize the quantity:

(6) $$\begin{array}{c} I\left(\overset{\leftarrow }{x}:R\right)+\alpha I\left(\overset{\leftarrow }{x}:\vec{x}|R\right) \end{array}$$

for some α>0. The first term captures the information that the model carries about the observed dynamics $\overset{\leftarrow }{x}$, the second term captures the information that the past dynamics carry about the future given the filter imposed by the model *R*, and the metaparameter α weights the importance of each term towards the global optimization.

We will evaluate our probabilistic grammars in a similar (yet slightly different) fashion. For our method of choice, we first acknowledge that we are facing a Pareto, or Multi-Objective Optimization (MOO) problem [23,24,25]. In this kind of problem we attempt to minimize or maximize different traits of the model simultaneously. Such efforts are often in conflict with each other. In our case, we want to make our models as simple as possible, but in that simplicity we ask that they retain as much of their predictive power as possible. We will quantify how different grammars perform in both these regards, and rank them accordingly. MOO problems rarely present global optima, i.e., we will not be able to find the best grammar. Instead, MOO solutions are usually embodied by Pareto-optimal trade-offs. These are collections of designs that cannot be improved in both optimization targets simultaneously. In our case these will be grammars that cannot be made simpler without losing some accuracy in their description of a text, or that cannot be made more accurate without making them more complicated.

The solutions to MOO problems are connected with statistical mechanics [25,26,27,28,29]. The geometric representation of the optimal trade-off reveals phase transitions (similar to the phenomena of water turning into ice or evaporating promptly with slight variations of temperature around 0 or 100 degrees Celsius) and critical points. In our case, Pareto optimal grammars would give us a collection of linguistic descriptions that simultaneously optimize how simply language rules can become while retaining as much of their explanatory power as possible. The different grammars along a trade-off would become optimal descriptions at different levels, depending on how much detail we wish to track about a corpus. Positive (second order) phase transitions would indicate salient grammars that are adequate descriptions of a corpus at several scales. Negative (first order) phase transitions would indicate levels at which the optimal description of our language changes drastically and very suddenly between extreme sets of rules. Critical points would indicate the presence of somehow irreducible complexity in which different descriptions of a language become simultaneously necessary, and aspects included in one description are not provided by any other. Although critical points seem a worst-case scenario towards describing language, they are a favorite of statistical physics. Systems at a critical point often display a series of desirable characteristics, such as versatility, enhanced computational abilities, and optimal handling of memory [30,31,32,33,34,35,36,37,38].

In Section 2 we explain how we infer our π and Ψ (i.e., our abstract “grammatical classes” and associated grammars), and the mathematical methods used to quantify how simple and accurate they are. In Section 3, we present some preliminary results, always keeping in mind that this paper is an illustration of the intended methodology. More thorough implementations will follow in the future. In Section 4, we reflect about the insights that we might win with these methods, how they could integrate more linguistic aspects, and how they could be adapted to deal with the complicated, hierarchical nature of language.

## 2. Methods

### 2.1. Corpus Description and Preparation

We took a sample of 49 newspaper articles from the Corpus of Contemporary American English [39]. The articles were selected such that they did not contain foreign (non-English) words or symbols. We substituted by a period every punctuation mark that indicated the end of a sentence and removed any other punctuation mark except for the apostrophes indicating a contraction (e.g., “don’t”) or a genitive (e.g., “someone’s”). Ideally, we would like to use raw texts and see Pareto optimal grammars emerging from them. These should also include instructions about how alien symbols or words (loosely speaking, any items that are not proper of English language, e.g., french terms, accent marks, etc.) are treated. However, these are rather minor details. Effective grammars should specify first how its own words are articulated.

Our more basic level of analysis will already be a coarse-grained one. Again, ideally, we would present our methods with texts in which each word is explicitly expelled out. Our blind techniques should then infer grammatical classes (if any were useful) based on how different words correlate. For example, we expect that our blind methods would be able, at some point, to group all nouns together based on their syntactic regularities. While this is possible, it is very time- and resource-consuming for the demonstration intended here. Therefore, we preprocessed our corpus using Python’s Natural Language Processing Toolkit [40] to map every word into one of the $N_{G}=34$ grammatical classes shown in Table 1. We then substituted every word in the corpus by its grammatical class. The resulting texts constitute the symbolic dynamics that we analyze.

### 2.2. Word Embeddings and Coarse-Graining

We would like to explore the most general grammars possible. However, as advanced above, to make some headway we restrict ourselves to grammar models that encode a tongue’s rules in a probabilistic way, telling us how likely it is that words follow each other in a text. Even in this narrower class there is an inscrutably large number of possibilities depending, e.g., on how far back we look into a sentence to determine the next word’s likelihood, on whether we build intermediate phrases to keep track of the symbolic dynamics in a hierarchical way, etc. Here, we only attempt to predict the next word given the current one. We will also restrict ourselves to maximum entropy (*MaxEnt*) models, which are the models that introduce less further assumptions provided a series of observations [37,41,42,43,44,45,46,47,48,49]. We explain these kind of models in the next subsection. First, we need to introduce some notation and a suitable encoding of our corpus so we can manipulate it mathematically.

We use a one-hot embedding, which substitutes each word in a text by a binary string that consists of all zeros and exactly one 1. The position of the 1 indicates the class of word that we are dealing with. Above, we illustrated several levels of coarse-graining. In a very fundamental one, each word represents a class of its own. Our vocabulary in the simple example sentence “green colorless ideas sleep furiously” consists of

(7) $$\begin{array}{c} \chi^{words}\equiv \left\{ideas,sleep,green,colorless,furiously\right\} \end{array}$$

which in its binary form becomes

(8) $$\begin{array}{c} {\tilde{\chi }}^{words}=\left\{10000,01000,00100,00010,00001\right\}. \end{array}$$

We also illustrated a level of coarse-graining in which nouns and verbs are retained, but all other words are grouped together in a third category (Equation (4)). The corresponding vocabulary

(9) $$\begin{array}{c} \chi \equiv \{noun,verb,cat_{3}\} \end{array}$$

becomes, through the one-hot embedding:

(10) $$\begin{array}{c} \tilde{\chi }=\left\{100,010,001\right\}. \end{array}$$

Throughout this paper, we will note by $\chi^{\lambda }$ the vocabulary (set of unique symbols) at a description level λ, and we will refer by ${\tilde{\chi }}^{\lambda }$ to its one-hot representation. We will name $c_{j}^{\lambda }\in \chi^{\lambda }$, with $j\in \{1,\ldots ,N^{\lambda }\}$, to each of the $N^{\lambda }$ unique symbols at description level λ. Each of these symbols stands for an abstract class of words, which might or might not correspond to actual grammatical classes in the standard literature. The binary representation of each class is correspondingly noted by $\sigma_{j}^{\lambda }\in {\tilde{\chi }}^{\lambda }$.

**Figure 2:**
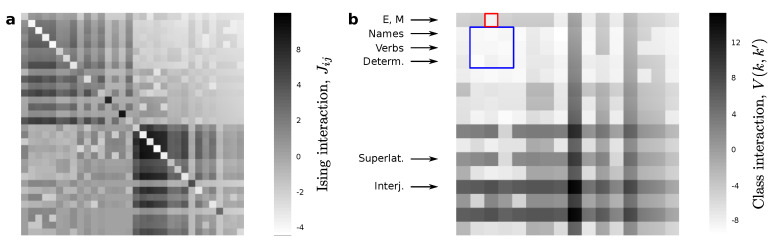
Interactions between spins and word classes. (**a**) A first crude model with spins encloses more information than we need for the kind of calculations that we wish to do right now. (**b**) A reduced version of that model gives us an interaction energy between words or classes of words. These potentials capture some non-trivial features of English syntax, e.g., the existential “there” in “there is” or modal verbs (marked E and M respectively) have a lower interaction energy if they are followed by verbs. Interjections present fairly large interaction energy with any other word, perhaps as a consequence of their independence within sentences.

**Figure 3:**
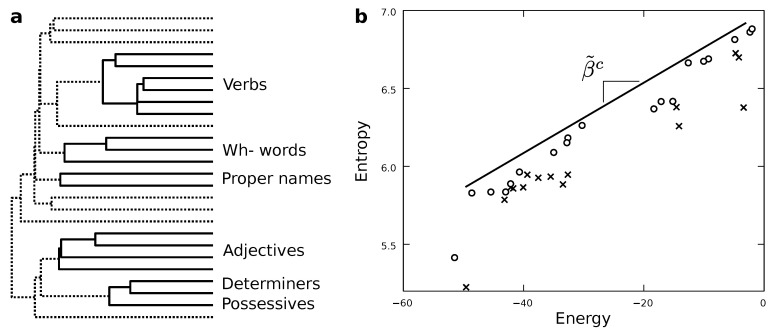
Pareto optimal maximum entropy models of human language. Among all the models that we try out, we prefer those Pareto optimal in energy minimization and entropy maximization. (**a**) These reveal a hierarchy of models in which different word classes group up at different levels. The clustering reveals a series of grammatical classes that belong together owing to the statistical properties of the symbolic dynamics, such as possessives and determiners which appear near to adjectives. (**b**) A first approximation to the Pareto front of the problem. Future implementations will try out more grammatical classes and produce better quality Pareto fronts, establishing whether phase transitions or criticality are truly present.

To explore models of different complexity we start with all the grammatical classes outlined in Table 1 and proceed by lumping categories together. We will elaborate a probabilistic grammar for each level of coarse-graining. Later, we will compare the performance of all descriptions. In lumping grammatical classes together there are some choices more effective than others. For example, it seems wise to group comparative and superlative adverbs earlier than nouns and verbs. We expect the former to behave more similarly than the later, and therefore to lose less descriptive power when treating both comparative and superlative adverbs as one class. In future versions of this work, we intend to explore arbitrary lumping strategies. Here, to produce results within a less demanding computational framework, we use an informed shortcut. We build the maximum entropy model of the least coarse-grained category (which, again, in this paper consists of the grammatical classes in Table 1). Through some manipulations explained below, this model allows us to extract correlations between a current word and the next one (illustrated in Figure 2). These correlations allow us to build a dendogram (Figure 3a) based on how similarly different grammatical classes behave.

This dendogram suggests an order in which to merge the different classes, which is just a good guess. There are many reasons why the hierarchy emerging from the dendogram might not be the best coarse-graining. We will explore more exhaustive possibilities in the future. In any case, this scheme defines a series of functions $\pi^{\lambda }$ (which play the role of π in Figure 1) that map the elements of the most fine-grained vocabulary $\chi^{0}\equiv \chi^{grammar}$ (as defined by the classes in Table 1) into a series of each time more coarse-grained and abstract categories $\chi^{\lambda }$, with $\lambda =1,\ldots ,N_{G}-1$ indicating how many categories have been merged at that level.

### 2.3. Maximum-Entropy Models

To build the MaxEnt model at a given level λ of coarse-graining, we substitute every word in our corpus by its binary representation. Our text then becomes a binary string. For example, with the coarse-graining in which nouns and verbs are kept, and all other words are abstracted into $cat_{3}$, we have

(11) $$\begin{array}{c} \mathrm{green}\;\;\mathrm{colorless}\;\;\mathrm{ideas}\;\;\mathrm{sleep}\;\;\mathrm{furiously}⟶001\;\;001\;\;100\;\;010\;\;001. \end{array}$$

We indicate the *i*-th word in a text by w(i). Its grammatical class in the description level λ is noted:

(12) $$\begin{array}{c} c^{\lambda }\left(i\right)\equiv \pi^{\lambda }\left(w\left(i\right)\right), \end{array}$$

and its binary representation:

(13) $$\begin{array}{c} \sigma^{\lambda }\left(i\right)\equiv {\tilde{\pi }}^{\lambda }\left(w\left(i\right)\right). \end{array}$$

Both mappings $\pi^{\lambda }$ and ${\tilde{\pi }}^{\lambda }$ contain the same information, and both of them play the role of π in Figure 1. Note that $c^{\lambda }\left(i\right)=c_{j}^{\lambda }$ for some *j*, and that although $i\in \{1,\ldots ,N_{w}\}$ indexes words as they happen in a text (of length $N_{w}$), $j\in \{1,\ldots ,N^{\lambda }\}$ indexes unique grammatical classes in $\chi^{\lambda }$. Each binary representation consists of $N^{\lambda }$ bits. When necessary, we will use a subindex *k* to label $\sigma_{j,k}^{\lambda }$ as the *k*-th bit of the *j*-th class’s binary representation at a given coarse-graining level λ.

We next produce binary samples that include each word and the one next to it in a text: $\langle \sigma^{\lambda }\left(i\right)|\sigma^{\lambda }\left(i+1\right)\rangle$, where $\langle \cdot |\cdot \rangle$ indicates concatenation. Thus, the coarse-grained sentence from Equation (11) yields the samples:

(14) $$\begin{array}{c} \{001001,001100,100010,010001\}. \end{array}$$

Each sample has size $2N^{\lambda }$ (when needed, the index *k* over bits will also label positions from 1 to $2N^{\lambda }$). Large corpora will produce huge collections of such samples. We can summarize these collections by giving the empirical frequency $F\left(\langle \sigma_{j}^{\lambda }|\sigma_{j^{\prime }}^{\lambda }\rangle \right)$ with which each of the ${\left(N^{\lambda }\right)}^{2}$ possible bit strings with length $2N^{\lambda }$ shows up. These collections behave as samples of what is known as spin glasses in statistical mechanics. We have powerful mathematical tools to infer MaxEnt models for spin glasses – therefore all these efforts.

## 3. Results

Using the methodology described above, we have coarse-grained the words of a written corpus, first, into the 34 grammatical classes shown in Table 1. This process is illustrated by Equation (2). The resulting symbolic series was binarized to create samples akin to spin glasses, a well studied model from statistical mechanics that allows us to use powerful mathematical tools on our problem. This process was then repeated at several levels of coarse graining as words were further lumped into abstract grammatical categories (e.g., as in Equation (4)). At each level of description, the inferred spin glass model plays the role of a grammar that constrains, in a probabilistic fashion, how word classes can follow each other in a text. These mathematical tools from spin glass theory allow us to test grammars from different description levels against each other as will become clear now.

In spin glasses, a collection of little magnets (or spins) is arranged in space. We say that a magnet is in state σ=1 if its north pole is pointing upwards and in state σ=−1 if its pointing downwards (these are equivalent to the 1s and 0s in our word samples). Two of these little magnets interact through their magnetic fields. These fields build up a force that tends to align both spins in the same direction, whichever it is, just as two magnets in your hand try to fall along a specific direction with respect to each other. On top of this, the spins can interact with an external magnetic field—bringing in a much bigger magnet which orientation cannot be controlled. This external field tends to align the little spins along its fixed, preferred direction. Given the spin states $\sigma_{1}$ and $\sigma_{2}$, the energy of their interaction with the external magnetic field and with each other can be written as

(15) $$\begin{array}{ccc} E(\sigma_{1},\sigma_{2}) & = & -\frac{1}{2}\left(2h_{1}\sigma_{1}+\sigma_{1}J_{12}\sigma_{2}+\sigma_{2}J_{21}\sigma_{1}+2h_{2}\sigma_{2}\right)\\ & = & -\frac{1}{2}\left(J_{11}\sigma_{1}+\sigma_{1}J_{12}\sigma_{2}+\sigma_{2}J_{21}\sigma_{1}+J_{22}\sigma_{2}\right). \end{array}$$

$J_{12}$ and $J_{21}$ (with $J_{12}=J_{21}$) denote the strength of the interaction between the spins, and $J_{11}\equiv 2h_{1}$ and $J_{22}=2h_{2}$ denote the interaction of each spin with the external field. The terms $h_{1}$ and $h_{2}$ are also known as biases. If the spins are aligned with each other and with the external field, the resulting energy is the lowest possible. Each misalignment increases the energy of the system. In physics, states with less energy are more probable. Statistical mechanics allows us to write precisely the likelihood of finding this system in each of its four ({1,1}, {1,−1}, {−1,1}, and {−1,−1}) possible states:

(16) $$\begin{array}{ccc} P(\sigma_{1},\sigma_{2}) & = & \frac{e^{-\beta E(\sigma_{1},\sigma_{2})}}{Z}, \end{array}$$

where β=1/T is the inverse of the temperature. The term

(17) $$\begin{array}{ccc} Z & = & e^{-\beta E(1,1)}+e^{-\beta E(1,-1)}+e^{-\beta E(-1,1)}+e^{-\beta E(-1,-1)}\\ & = & \sum_{\sigma_{1},\;\sigma_{2}=\pm 1}e^{-\beta E(\sigma_{1},\sigma_{2})} \end{array}$$

is known as the partition function and is a normalizing factor that guarantees that the probability distribution in Equation (16) is well defined.

Back to our text corpus in its binary representation, we know the empirical frequency $F\left(\langle \sigma_{j}^{\lambda }|\sigma_{j^{\prime }}^{\lambda }\rangle \right)$ with which each of the possible spin configurations shows up—we just need to read it from our corpus. We can treat our collection of 0s and 1s as if they were ±1 samples of a spin glass, and attempt to infer the $\beta^{\lambda }$ and $J^{\lambda }$ which (through a formula similar to Equation (16)) more faithfully reproduce the observed sample frequencies. The superindex in $\beta^{\lambda }$ and $J^{\lambda }$ indicates that they will change with the level of coarse-graining. Inferring those $\beta^{\lambda }$ and $J^{\lambda }$ amounts to finding the MaxEnt model at that coarse-grained level. As advanced above, MaxEnt models are convenient because they are the models that introduce less extra hypotheses given some observations. In other words, if we infer the MaxEnt model for some λ, any other model with the same coarse-graining would be introducing spurious hypotheses that are not suggested by the data. To infer MaxEnt models, we used Minimum Probability Flow Learning (MPFL [50]), a fast and reliable method that infers the $J^{\lambda }$ given a sufficiently large sample.

Each grammatical class is represented by $N^{\lambda }$ spins at the λ-th coarse-graining. This implies, as we know, that our samples consists of $2N^{\lambda }$ spints. MPFL returns a matrix $J^{\lambda }$ of size $2N^{\lambda }\times 2N^{\lambda }$. This matrix embodies our abstract, probabilistic grammar (and plays the role of Ψ in Figure 1). Each entry $J_{kk^{\prime }}^{\lambda }$ of this matrix tells us the interaction energy between the *k*-th and $k^{\prime }$-th bits in a sample (with $k,k^{\prime }=1,\ldots ,2N^{\lambda }$). However, each grammatical class is represented not by one spin, but by a configuration of spins that has only one 1. To obtain the interaction energies between grammatical classes (rather than between spins), we need to compute

(18) $$\begin{array}{ccc} V^{\lambda }\left(c_{j}^{\lambda },c_{j^{\prime }}^{\lambda }\right) & = & \frac{1}{2}\sum_{k,k^{\prime }}\sigma_{j,k}^{\lambda }J_{kk^{\prime }}^{\lambda }\sigma_{j^{\prime },k^{\prime }}^{\lambda }. \end{array}$$

This energy in turn tells us the frequency with which we should observe each pair of words according to the model:

(19) $$\begin{array}{ccc} P^{\lambda }\left(\langle c_{j}^{\lambda }|c_{j^{\prime }}^{\lambda }\rangle \right) & = & \frac{1}{Z^{\lambda }}e^{\beta V^{\lambda }\left(c_{j}^{\lambda },c_{j^{\prime }}^{\lambda }\right)}. \end{array}$$

We inferred MaxEnt models for the more fine-grained level of description ($\chi^{0}$ as given by the grammatical classes in Table 1), as well as for every other intermediate level $\chi^{\lambda }$. Figure 2a shows the emerging spin-spin interactions for l=15, which consists of only 19 (versus the original 34) grammatical classes. This matrix presents a clear box structure:

(20) $$\begin{array}{ccc} J^{\lambda } & = & \left[\begin{array}{cc} 2h^{\lambda } & {\vec{\partial }}^{\lambda }\\ ine{\overset{\leftarrow }{\partial }}^{\lambda } & 2{\bar{h}}^{\lambda } \end{array}\right]. \end{array}$$

The diagonal blocks ($2h^{\lambda }$ and $2{\bar{h}}^{\lambda }$) represent the interactions between all spins that define, separately, the first and second words in each sample. As our corpus becomes infinitely large, $h^{\lambda }\to {\bar{h}}^{\lambda }$. These terms do not capture the interaction between grammatical classes. In the spin-glass analogy, they are equivalent to the interaction of each word with the external magnet that biases the presence of some grammatical classes over others. Such biases affect the frequencies $P^{\lambda }\left(c_{j}^{\lambda }\right)$ with which individual classes show up, but not the frequency with which they are paired up. Therefore, the $h^{\lambda }$ and ${\bar{h}}^{\lambda }$ are not giving us much syntactic information.

More interesting for us are the interaction terms stored in ${\vec{\partial }}^{\lambda }$ and ${\overset{\leftarrow }{\partial }}^{\lambda }$. The inference method used guarantees that ${\vec{\partial }}^{\lambda }={\left({\overset{\leftarrow }{\partial }}^{\lambda }\right)}^{T}$. It is from these terms that we can compute the part of $V^{\lambda }\left(c_{j}^{\lambda },c_{j^{\prime }}^{\lambda }\right)$ (shown in Figure 2b) that pertains to pairwise interaction alone (i.e., the energy of the spin system when we discount the interaction with the external field). $V^{\lambda }\left(c_{j}^{\lambda },c_{j^{\prime }}^{\lambda }\right)$ encodes the energy of two word classes when they are put next to each other in a text. The order in which words appear after each other is relevant, therefore that matrix is not symmetric. These energies reflect some of the rules of English. For example, the first row (labeled “E, M”) is a class that has lumped together the existential “there” (as in “there is” and “there are”) with all modal verbs. These tend to be followed by a verb in English, thus the matrix entry coding for $\langle “E,M^{”}|“verb^{”}\rangle$ (marked in red) is much lower than most entries for any other $\langle “E,M^{”}|\cdot \rangle$. The blue square encompasses verbs, nouns, and determiners. Although the differences there are very subtle, the energies reflect that it is more likely to see a noun after a determiner and not the other way around, and also that it is less likely to see a verb after a determiner.

It is not straightforward to compare all energies because they are affected by the raw frequency with which pairs of words show up in a text. In that sense, our corpus size might be sampling some pairings insufficiently so that their energies do not reflect proper English use. On the other hand, classes such as nouns, verbs, and determiners happen so often (and so often combined with each other) that they present very low energies as compared with other possible pairs. This makes the comparison more difficult by visual inspection.

It is possible to use $V^{\lambda }\left(c_{j}^{\lambda },c_{j^{\prime }}^{\lambda }\right)$ to generate a synthetic text ${\tilde{T}}^{\lambda }$ and evaluate its energy $E^{0}\left({\tilde{T}}^{\lambda }\right)$ using the most fine-grained model $J^{0}$. If the coarse-grained model $V^{\lambda }\left(c_{j}^{\lambda },c_{j^{\prime }}^{\lambda }\right)$ retains a lot of the original structure, the generated text will fit gracefully in the rules dictated by $J^{0}$—just as magnets falling into place. Such texts would present very low energy when evaluated by $J^{0}$. If the coarse-grained model has erased much of the original structure, the synthetic text will present odd pairings. These would feel similar to magnets that we are forcing into a wrong disposition, therefore resulting in a large energy when $J^{0}$ is used. In other words, this energy reflects how accurate each coarse-grained model is.

That accuracy is one of the targets in our MOO problem, in which we attempt to retain as much information as possible with models as simple as possible. To quantify that second target, simplicity, we turn to entropy. The simplest model possible generates words that fall in either class of $\chi^{0}$ randomly and uniformly, thus presenting the largest entropy possible. More complex models, in their attempt to remain accurate, introduce constraints as to how the words in the coarse-grained model must be mapped back into the classes available in $\chi^{0}$. That operation would be the reverse of $\pi^{\lambda }$. This reverse mapping, however, cannot be undone without error because the coarse-graining erases information. Entropy measures the amount of information that has been erased, and therefore how simple the model has been made.

Figure 3b shows the energy $E^{0}\left(T^{\lambda }\right)$ and entropy $S^{0}\left(T^{\lambda }\right)$ for synthetic texts generated with the whole range of coarse-grainings explored. In terms of Pareto optimality, we expect our models to have as low an energy as possible while having the largest entropy compatible with each energy—just as thermodynamic systems do. Such models would simultaneously optimize their simplicity and accuracy. Within the sample, some of these models are Pareto dominated (crosses in Figure 3b) by some others. This means that for each of those models at least some other one exists that is simpler and more accurate at the same time. These models are suboptimal regarding both optimization targets, so we do not need to bother with them.The non-dominated ones (marked by circles in Figure 3b) capture better descriptions in both senses (accuracy and simplicity). They are such that we cannot move from one to another without improving an optimization target and worsening the other. They embody the optimal trade-off possible (of course, limited by all the approximations made in this paper), and we cannot choose a model over the others without introducing some degree of artificial preference either for simplicity or accuracy.

In statistical mechanics the energy and entropy of a system are brought together by the free energy:

(21) $$\begin{array}{c} F=E-\hat{T}S=E-S/\hat{\beta }. \end{array}$$

Here, $\hat{T}$ plays a role akin to a temperature and $\hat{\beta }$ plays the role of its inverse. We noted $\hat{\beta }\neq \beta$ to indicate that these temperature and inverse temperature are different from the ones in Equation (19). Those temperatures control how often a word shows up given a model, whereas $\hat{\beta }$ controls how appropriate each level of description is. When $\hat{\beta }$ is low (and $\hat{T}$ is large), a minimum free energy in Equation (21) is attained by maximizing the entropy rather than minimizing the energy. This is, low $\hat{\beta }$ selects for simpler descriptions. When $\hat{\beta }$ is large (and $\hat{T}$ is small), we prefer models with lower energy, i.e., higher accuracy.

By varying $\hat{\beta }$ we visit the range of models available, i.e., we visit the collection of Pareto optimal grammars (circles in Figure 3b). In statistical mechanics, by varying the temperature of a system we visit a series of states of matter (this is, we put, e.g., a glass of water at different temperatures and observe how its volume and pressure change). At some relevant points, called phase transitions, the states of matter change radically, e.g., water freezes swiftly at 0 degrees Celsius, and evaporates right at 100 degrees Celsius. The geometry of Pareto optimal states of matter tells us when such transitions occur [25,26,27,28,29].

Similarly, the geometric disposition of Pareto optimal models in Figure 3b tells us when a drastic change in our best description is needed as we vary $\hat{\beta }$. Relevant phase transitions are given by cavities and salient points along the Pareto optimal solutions. In the first approach, we observe several cavities. More interestingly, perhaps, is the possibility that our Pareto optimal models might fall along a straight line; one has been added as a guideline in Figure 3b. Although there are obvious deviations from it, such description might be feasible at large. Straight lines in this plot are interesting because they indicate the existence of special critical points [28,37,46,47,48]. In the next section, we discuss what criticality might mean in this context.

## 4. Discussion

In this paper, we study how different hierarchical levels in the description of human language are entangled with each other. Our work is currently at a preliminary stage, and this manuscript aims at presenting overall goals and a possible methodological way to tackle relevant questions. Some interesting results are presented as an illustration and discussed in this section to exemplify the kind of debate that this line of research can spark.

Our work puts forward a rigorous and systematic framework to tackle the questions introduced above, namely, what levels of description are relevant to understand human language and how do these different descriptions interact with each other. Historically, we have answered these questions guided by intuition. Some aspects of language are so salient that they demand a sub-field of their own. Although this complexity and interconnectedness is widely acknowledged, its study is still fairly compartmentalized. The portray of language as a multilayered network system is a recent exception [8], as it is the notable and lasting effort by Christiansen et al. [9,10] to link all scales of language production, development, and evolution in a unified frame.

We generated a collection of models that describe a written English corpus. These models trade optimally a decreasing level of accuracy by increasing simplicity. By doing so, they gradually lose track of variables involved in the description at more detailed levels. For example, as we saw above, the existential “there” is merged with modal verbs. Indeed, these two classes were lumped together before the distinction between all other verbs was erased. Although those grammatical classes are conceptually different, our blind methodology found convenient to merge them earlier in order to elaborate more efficient compact grammars.

Remaining as accurate as possible while becoming as simple as possible is a multi-objective optimization problem. The conflicting targets are captured by the energy and entropy that artificial texts generated by a coarse-grained model have when evaluated at the most accurate level of description. We could have quantified these targets in other ways (e.g., counting the number of grammatical classes to quantify complexity, and measuring similarity between synthetic and real texts for accuracy). Those alternative choices should be explored systematically in the future to understand which options are more informative. Our choices, however, make our results easy to interpret in physical terms. For example, improbable (unnatural) texts have high energies in any good model.

The grammars that optimally trade between accuracy (low energy) and simplicity (high entropy) conform the Pareto front (i.e., the solution) of the MOO problem. Its shape in the energy-entropy plane (Figure 3) is linked to phase transitions [25,26,27,28,29]. According to this framework, we do not find evidence of a positive (second order) phase transition. What could such a transition imply for our system? The presence of a positive phase transition in our data would suggest the existence of a salient level of description capable of capturing a large amount of linguistic structure in relatively simple terms. For example, if a unique grammatical rule would serve to connect words together disregarding of the grammatical classes in which we have split our vocabulary. We would expect that to be the case, e.g., if a single master rule such as merge would serve to generate all the complexity of human language without further constraints arising. This does not seem to be the case. However, this does not rule out the existence of the relevant merge operation, nor does it deny its possible fundamental role. Indeed, Chomsky proposes that merge is the fundamental operation of syntax, but that it leaves the creative process of language underconstrained [51,52,53]. As a result, actual implementations (i.e., real languages) see a plethora of further complexities arising in a phenomena akin to symmetry breaking.

The presence of a negative (first order) phase transition would acknowledge several salient levels of description needed to understand human language. These salient descriptions would furthermore present an important gap separating them. This would indicate that discrete approaches would be possible to describe language without missing any detail by ignoring the intermediate possibilities. If that were the case, we would still need to analyze the emerging models and look at similarities between them to understand whether both models capture a same core phenomenology at two relevant (yet distant) scales; or whether each model focuses on a specific, complementary aspect that the other description has no saying about. Some elements in Figure 3b are compatible with this kind of phase transition.

However, the disposition of several Pareto optimal grammars along a seemingly straight line rather suggests the existence of a special kind of critical phenomenon [28,37,46,47,48]. Criticality is a worst-case scenario in terms of description. It implies that there is no trivial model, nor couple of models, nor relatively small collection that can capture the whole of linguistic phenomenology at any level. A degenerate number of descriptions is simultaneously necessary, and elements trivial in a level can become cornerstones of another. Also, potentially, constraints imposed by a linguistic domain (e.g., phonology) can penetrate all the way and alter the operating rules of other domains (e.g., syntax or semantics). We can list examples of how this happens in several tongues (such as the case of determiners “a” and ‘an’ in English mentioned above). The kind of criticality suggested by our results would indicate that such intrusions are the norm rather than the exception. Note that this opportunistic view of grammar appears compatible with Christiansen’s thesis that language evolved, as an interface, to make itself useful to our species, necessarily exploiting all kinds of hacks along its way [9].

Zipf’s law is a notable distribution in linguistics [54,55]. It states that the *n*-th most abundant word in a text shows up with a frequency that is inversely proportional to that word’s rank (i.e., *n*). The presence of this distribution in linguistic corpora has been linked to an optimal balance between communicative tensions [54,56,57]. It has also been proved mathematically that Zipf’s law is an unavoidable feature of open-ended evolving systems [58]. Languages and linguistic creativity are candidates to present open-ended evolution. Could this open-endedness be reflected also in the diversity of grammatical rules that form a language? Could we expect to find a power-law in the distribution of word combinations with a given energy? If that were the case, Bialek et al. [37,47] proved mathematically that the relationship between energy and entropy of such grammars must be linear and therefore critical. In other words, our observation of criticality in this work, if confirmed, would be a strong hint (yet not sufficient) that the relevant Zipf distribution may also be lurking behind grammars derived empirically from written corpora.

Numerous simplifications were introduced to produce the preliminary results in this paper. We started our analysis with words that have already been coarse-grained into 34 grammatical classes, barring the emergence of further intermediate categories dictated, e.g., by semantic use. We know that semantic considerations can condition combinations of words, such as what verbs can be applied to what kinds of agents [59]. The choice of words as units (instead of letters or syllables) is another limiting factor. Words are symbols whose meanings do not depend on physical correlates with the objects signified [60]. In that sense, their association to their constituent letters and phonems is arbitrary. Their meaning is truly emergent and not rooted in their parts. Introducing letters, syllables, and phonetics in our analysis might reveal and allow us to capture that true emergence.

To do this it might be necessary to work with hierarchical models that allow correlations beyond the next and previous words considered here. This kind of hierarchy, in general, is a critical aspect of language [53] that our approach should capture in due time. We have excluded it in this work to attain preliminary results in a reasonable time. Although hierarchical models are likely to be more demanding (in computational terms), they can be parsimoniously incorporated in our framework. A possibility is to use epsilon machines [61,62,63], which naturally lump together pieces of symbolic dynamics to find out causal states. These causal states act as shielding units that advance a symbolic dynamics in a uniquely determined way—just like phrases or sentences provide a sense of closure at their end, and direct the future of a text in new directions.

## Figures and Tables

**Table 1:** Grammatical classes present in the most fine-grained level of our corpora.

Conjunction	Adverb
Cardinal number	Adverb, comparative
Determiner	Adverb, superlative
Existential there	to
Preposition	Interjection
Adjective	Verb, base form
Adjective, comparative	Verb, past tense
Adjective, superlative	Verb, gerund or present participle
Modal	Verb, past participle
Noun, singular	Verb, non-3rd person singular present
Noun, plural	Verb, 3rd person singular present
Proper noun, singular	Wh-determiner
Proper noun, plural	Wh-pronoun
Predeterminer	Possessive wh-pronoun
Possessive ending	Wh-adverb
Personal pronoun	None of the above
Possessive pronoun	‘.’
